# Declining demands or constraining supply? Explaining the trend of care home entry in England 2001–2021

**DOI:** 10.1093/geronb/gbag104

**Published:** 2026-06-08

**Authors:** Jingwen Zhang, Maria Petrillo, Matthew R Bennett, Gwilym Pryce

**Affiliations:** Department of Sociology, School of Social Sciences, The University of Manchester, Manchester, United Kingdom; ESRC Centre for Care, United Kingdom; ESRC Centre for Care, United Kingdom; School of Sociological Studies, Politics and International Relations, Centre for International Research on Care, Labour and Inequalities (CIRCLE), University of Sheffield, Sheffield, United Kingdom; ESRC Centre for Care, United Kingdom; School of Social Policy and Society, University of Birmingham, Birmingham, United Kingdom; ESRC Centre for Care, United Kingdom; School of Economics, University of Sheffield, Sheffield, United Kingdom; (Social Sciences Section)

**Keywords:** Long-term care, Institutional care, Nonlinear decomposition, Nursing home

## Abstract

**Objectives:**

Despite population aging, care home residency in England has declined over time. However, little is known about the drivers of this decline, particularly the role of supply-side factors. Guided by the Andersen Behavioral Model, this study examines how predisposing, enabling, need, and supply-side contextual factors are associated with care home entry and identifies the mechanisms underlying the decline in institutional care use over time.

**Methods:**

Using newly linked data from the ONS Longitudinal Study 2001–2021 and the Care Quality Commission care directory (*n *= 89,122), we estimated logistic regression models to identify key predictors of care home entry. We then applied a nonlinear decomposition to quantify the contributions of compositional changes and changing associations to the declining trends over time.

**Results:**

The rates of people aged 65 years and older moving from private households to care homes declined from 5.5% in 2001–2011 to 4.0% in 2011–2021. Poor health remained the strongest predictor of care home entry in both periods, while homeownership, being married, and lower local care home bed supply were associated with reduced likelihood of admission. Decomposition results showed that 85.3% of the decline was explained by compositional changes, primarily improvements in population health, alongside reduced supply of care home beds and shifting ethnic composition.

**Discussion:**

The findings indicate that promoting healthy aging remains central to delaying institutionalization, while declining institutional capacity raises concerns about future access and equity in long-term care provision. This study also offers insights for care home projections and effective resource allocation.

Care homes provide essential personal or specialist care and accommodation for people with complex medical conditions or those struggling to live independently (Burton et al., 2021). They are important long-term care (LTC) service providers, and sometimes the best or only option, for people who require the most intensive care. However, compared with home-based care, care home provision is substantially more expensive, with a weekly cost of £1,200–£1,800 per resident (Jones et al., 2025). To ensure the financial sustainability of the LTC system, the policy agenda in many aging societies has emphasized delaying people’s entry into care homes and encouraging the use of home and community-based services (Spasova et al., 2018).

Although the number of care home residents in England has long been projected to rise due to population aging (King et al., 2010; Kingston et al., 2022), recent data from the National Health Service (NHS) Digital show the admission rates to care homes declined from 658.5 per 100,000 population in 2014/15 to 538.5 in 2021/22 (NHS Digital, 2022). This phenomenon is not unique to the UK; several European countries, including the Netherlands and Cyprus, also witnessed a fall in the institutional care use rates in recent decades (De Meijer et al., 2015; Anderson & Molinuevo, 2017). Despite extensive research on predictors of care home entry (Grundy & Glaser, 1997; Grundy & Jitlal, 2007; McCann et al., 2012), no study has yet examined the drivers behind England’s recent downward trend in institutional care use. While studies in other European countries have identified potential drivers of declining institutional care use (Alders et al., 2017; De Meijer et al., 2015), existing studies have only considered individual-level or demand-side factors, without assessing the specific contribution of supply-side and contextual factors, which are key to individuals’ LTC arrangements (Tomiak et al., 2000). Understanding the factors driving the decline in care home residency is critical for policymakers, as different underlying causes (e.g., reduced care needs, shifting social norms around care, or diminished supply of care resources) carry different policy implications.

This study makes three novel contributions. First, using the most recent data from the Office for National Statistics Longitudinal Study (ONS-LS), a large and nationally representative sample, it provides the most up-to-date and robust evidence on trends in care home entry. The large sample size also allows us to trace care home entry among minority groups, such as ethnic minoritized groups and unpaid carers, which have been overlooked in existing research. Second, by linking ONS-LS and Care Quality Commission (CQC) administrative data (the regulator in England) for the first time, the study advances existing research by directly examining both demand- and supply-side determinants of care home use. Third, this is also the first study to apply nonlinear decomposition techniques to identify the key factors that explain the observed decline in institutional care use in the English context. By identifying the mechanisms driving this trend, the study offers critical insights for projecting future care home use and informing sustainable LTC policy and planning in England and comparable aging societies.

## Care home and LTC policy in England

In England, long-term care is primarily delivered through two types of care homes: residential homes and nursing homes. Residential homes provide accommodation, group activities, and personal care, such as washing and dressing. Nursing homes offer these services too, but also provide nursing care by qualified nurses, supporting people with more complex health and care needs (NHS UK, 2023).

Over the past three decades, LTC policy in England has increasingly prioritized “ageing in place,” supporting older people to remain at home or in the community and reducing reliance on more costly institutional options. The NHS and Community Care Act 1990 explicitly promoted a shift away from institutional care provision toward domiciliary alternatives (Grundy & Glaser, 1997). The 2001 National Service Framework for Older People further stressed the importance of providing intermediate care services at home to prevent premature or unnecessary admissions to residential care (Department of Health, 2001).

This policy shift has led to structural changes in the care home market and a redistribution of public funding for older people’s LTC. The role of local authorities shifted from direct providers to commissioners, and the private sector now dominates the care home sector, running 76% of all care home places in 2017 (LaingBuisson, 2017). The marketization of care homes has raised concerns about care home closures. Between 2011 and 2023, around 700 care homes closed annually due to voluntary exit driven by staff shortages and rising costs or quality failures (Bach-Mortensen et al., 2024). While institutional care has declined, the domiciliary care market has experienced continued growth. Between 2012 and 2023, the number of home care providers in England doubled from 5,600 to over 12,800, potentially diverting demand away from residential care (UK Homecare Association, 2016, 2024).

Social care in England is funded through local government revenue rather than directly through the national government budget (Foster & Harker, 2025). Access to publicly funded social care is means-tested, with eligibility restricted to individuals with the highest needs and assets below £23,250. In 2024/25, the average weekly cost of a local authority‑funded care home place for older people was £1,019, while self‑funders paid approximately 40% more (The King’s Fund, 2026). Although nearly 60% of care home residents receive public funding, the number of people being funded declined between 2005–2006 and 2012–2013 (Fernandez et al., 2013). With ongoing austerity, local authorities reduced their fee rates for care homes by over 6% from 2010–2011 to 2016–2017, pushing many providers to rely on self-funders. Consequently, nearly all new care homes are built in areas with a high concentration of self-funders, contributing to growing regional inequalities in care provision (Competition & Markets Authority, 2017).

## Conceptual framework

We identify factors associated with care home residence using the Andersen Behavioral Model of Health Services Use (Andersen & Davidson, 2013). This is the most widely used framework for analyzing healthcare service utilization, and it has been successfully adapted and applied to studies of LTC services (e.g., Wrotek & Kalbarczyk, 2023). The original model suggests that people’s use of health services is impacted by three sets of drivers: predisposing factors (e.g., demographic, social structure, and health beliefs), enabling factors (e.g., personal, family, and community resources), and needs (e.g., health and functional status). Recent developments of this model further emphasize the role of “contextual characteristics” (e.g., health or care organization, provider-related factors, and community characteristics) in shaping “individual determinants” and individuals’ access to services (Andersen & Davidson, 2013).

In the context of institutional care, *predisposing factors* are sociodemographic factors that underpin people’s perception of using care services, including age, sex, ethnicity, and caregiving history. Evidence has consistently shown that advanced age and being female are associated with a higher likelihood of care home admission due to functional decline and a greater likelihood of widowhood and reduced access to spousal care (Martikainen et al., 2009). Ethnic differences vary by national context and care system. In the UK, ethnic minoritized groups, especially Asian people, are underrepresented in care homes (Banks et al., 2006), largely due to concerns about cultural and religious appropriateness, socioeconomic disadvantages, and previous experiences of discrimination (Hossain et al., 2022). Care history influences individuals’ perceptions of aging, familiarity with community-based care, and anticipation of care needs, which may in turn shape their attitudes toward care homes (Capistrant, 2016; Hajek et al., 2017; Hu et al., 2023). However, empirical research examining care experiences and care home admission remains sparse.

*Enabling factors* (income/wealth, support from social networks, and availability of care facilities) facilitate access to institutional care. Although personal income/wealth enables people to self-fund care home services, findings on the economic gradient in institutional care utilization are mixed. Research in the UK and other European countries has mostly found that individuals with lower household incomes and non-homeowners are more likely to enter care homes (Abbing et al., 2023; McCann et al., 2012), partly because wealthier households can fund home-based alternatives or housing adaptations (Abbing et al., 2023). In England, most homeowners are not eligible for publicly funded care under the means-tested LTC system, further discouraging institutional entry. Living with a spouse or children, indicating available informal care, strongly predicts a lower likelihood of entering a care home in different national contexts (Martikainen et al., 2009; McCann et al., 2012). Few studies from the UK have examined how “contextual characteristics,” especially community care resources, shape an individual’s decision to move to a care home. Studies from Canada and the United States show that greater nursing home bed availability increases the likelihood of admission, while higher spending on home and community-based services reduces it (Muramatsu et al., 2007; Tomiak et al., 2000).

*Need factors, or care needs*, (general health, functional limitations, and perceived ability to live independently) are the primary determinants of service use. Most studies on determinants of institutional care have found that functional capacity and having dementia are the strongest positive predictors of using institutional care (Karmel et al., 2012; Wrotek & Kalbarczyk, 2023).

Understanding the contribution of need, predisposing, and enabling factors provides a foundation for explaining the declining trend of care home use in England. Two mechanisms have been key: (a) the distribution of these factors in the population changed (compositional effects); and (b) the relationships between these factors and care home utilization shifted, even when the distributions remained stable (coefficient effects).

Regarding compositional effects, evidence shows a relative decline in care needs. While the absolute number of people with disabilities increased by 3.9 million from 2012–2013 to 2022–2023 with population aging (Kirk-Wade et al., 2023), age-standardized disability prevalence, frailty levels, and years of life free of cognitive impairment have improved in England (Jagger et al., 2016; Old & Scott, 2023). Improvements in physical functioning may explain the reduced need for care homes. At the same time, austerity-driven reductions in public funding since 2010 may undermine the availability of institutional care facilities, the key contextual-level enablers for access to care homes. The number of care homes in England has declined since 1995, and closures were exacerbated by the COVID-19 pandemic (Ford & Smith, 2008; Nuffield Trust, 2025).

In terms of coefficient effects, these could be driven by changing LTC policies, technological advances in supporting independent living, shifting preferences for care services, and broader contextual influences, such as the COVID-19 pandemic. De Meijer et al. (2015) found that stricter eligibility thresholds were the primary driver of declining institutional care use in the Netherlands. In England, the deinstitutionalization of social care and the ongoing emphasis on “care in the community” may gradually shift social norms and expectations about care arrangements. However, there is limited longitudinal evidence and comparable data on changes in attitudes toward care settings over time in England. A study from the Netherlands, where policies similarly promote aging in place, used three proxy measures of the attractiveness of institutional care and found that declining attractiveness of institutional LTC relative to aging in place may explain 12.8%–19.2% of the reduction in institutional LTC use, providing support for the importance of social norms in shaping care arrangements (Alders et al., 2019). Meanwhile, increased availability of home adaptations and specialist older adult’s housing has strengthened the feasibility and desirability of aging in place (Local Government Association, 2022). Additionally, the high mortality rate in care homes during the COVID-19 pandemic further exposed long-standing issues in the sector, discouraging potential residents from moving into institutions.

Taken together, these trends have likely altered how need, predisposing, and enabling factors influence institutional care use and contribute to the declining care home use.

## The present study

Addressing a key gap in the existing literature, this study aims to answer two key research questions:

- How are individual- and contextual-level factors associated with moving into care homes in 2001–2011 and 2011–2021?
- What factors explain the trends in people moving into care homes from 2001–2011 to 2011–2021?

## Method

### Data

We used data from the ONS-LS for 2001−2021 (Office for National Statistics, 2025) and the CQC care directory. The ONS-LS consists of census records linked to life events data (births, deaths, and cancer registrations) of its approximately 1.3 million members. LS members are selected based on four confidential birthdates, resulting in a 1% representative sample of the population of England and Wales. The study began with the 1971 Census and has continued through the 1981, 1991, 2001, 2011, and 2021 censuses, providing up to 50 years of data on its sample members. New members enter the study either by being born on one of the four confidential birthdates or through immigration. They leave the study through death or emigration, although their records are retained in the dataset.

Aggregate numbers of care home beds by local authority were obtained from the Department of Health & Social Care, Palliative and End of Life Care Profile (Office for Health Improvement and Disparities, 2025). The original CQC care home-level data are publicly available from their official website.

Our analytic sample includes older adults aged 65 and over who were usual residents living in private households in England in 2001 ($t_{1}$) and still alive in 2011 ($t_{2}$), and an equivalent sample living in private households in 2011 ($t_{1}$) and alive in 2021 ($t_{2}$). In total, 441,731 and 395,809 usual residents present at $t_{1}\;$(i.e., 2001 and 2011, respectively) were followed up to $t_{2}$ (i.e., 10 years later) with complete census records. Although attrition was substantial: 49.38% during 2001−2011 and 45.63% during 2011−2021, approximately 90% of this attrition resulted from mortality. After limiting the sample to individuals aged 65 and over living in private households at the baseline period, the analytical sample size was reduced to 40,403 (2001−2011) and 48,890 (2011−2021). After excluding cases with missing values, the final analytical sample size was 89,122. The sample selection process and missing data patterns are detailed in Tables S1 and S2, respectively.

### Variables

*Care home residence* is derived from the types of communal establishments in 2011 and 2021. The subcategories considered as care homes include care homes with nursing (nursing homes) or without nursing (residential homes). Accurate identification of communal establishments is ensured using information from the Census Coverage Survey and cross-comparison with administrative data (Abbott, 2012). Nevertheless, previous research suggests that Census data may undercount smaller care homes (Banks et al., 2006).

*Predisposing facto*rs capture socio-demographic characteristics existing before the onset of care needs. In this study, we included age, sex, migration status (UK-born vs migrants), ethnicity (White vs ethnic minority), and unpaid care status (no care provided, 1–19 hr/week, 20–49 h/week, 50 hr/week). All predisposing factors are based on information at the start of each period.

*Enabling factors* include household type (not partnered, living with partner only, living with both partner and children, living with children only, other household type), marital status (not married, married/civil partnership), highest education qualification (no qualification, lower or other qualification, university degree), housing tenure (own outright, partly own, not own) and the rate of care home beds (per 100 people aged 75 and over), in the local authority where the respondent lived. Most of these factors are measured at the start of each period. In 2001, the highest education qualifications were recorded only for adults aged 16–74 years, meaning that education is missing for older people; therefore, we used education measured at the end of each period (2011/2021). Given that education is unlikely to change after age 65, this approach should not bias our results. The care home bed rates in 2011 and 2021 were calculated at the local authority level, measuring care resources available for local residents. We used care home information at the end of each period ($t_{2}$) rather than at the start $(t_{1}$), because the median length of stay in care homes is less than 2 years (Collingridge Moore et al., 2020). Therefore, care home resources at the end of each period ($t_{2}$) are a better proxy for resources available at the time of entry.

*Need factors* were measured by general health (“Very good,” “Good,” “Fair,” “Bad,” and “Very bad”) in 2011 and 2021. We used health status at the end of each period ($t_{2}$) because it is a better proxy for a person’s health at the time of admission than the equivalent measure at the start ($t_{1}$). Moreover, the questions on general health differ between 2001 and 2011, making direct comparison impossible.

### Analytical strategy

We first conducted a descriptive analysis, calculating the proportions of older people living in private households moving into care homes after 10 years, and comparing the distribution of predisposing, enabling, and need factors in 2001−2011 and 2011−2021, respectively. We then estimated logistic regression models to predict the probabilities of moving into a care home in 2011 and 2021, using mostly predictors from a decade earlier ($t_{1}$) and a few (i.e., education, health, and care home bed rates) from the respective Census years ($t_{2}$).

To decompose the drivers of changes in care home use, we used the Kitagawa–Oaxaca–Blinder type decomposition technique (Blinder, 1973; Kitagawa, 1955; Oaxaca, 1973). This method allows us to decompose the differences in care home use across years into two components: differences due to changes in characteristics over time (compositional effects) and differences due to changes in associations between care home use and characteristics (changing coefficient effects). However, the classical Kitagawa–Oaxaca–Blinder technique only applies to linear regressions. Given that the dependent variable is a binary variable and its relationships with explanatory variables are nonlinear, we employed a nonlinear decomposition method developed by Yun (2004) (see methodological details in the Supplementary Material).

The individual contribution of each covariate was derived using a “linearization” approach by approximating its relative contribution in a decomposition at the level of the linear predictor. The coefficients associated with categorical variables were normalized to address identification issues. Since individuals living in the same local authority may share similar characteristics (e.g., access to resources and local policies), standard errors were clustered at the local authority level.

## Results

### Trends in moving into a care home and their determinants from 2001 to 2021

**Figure 1:**
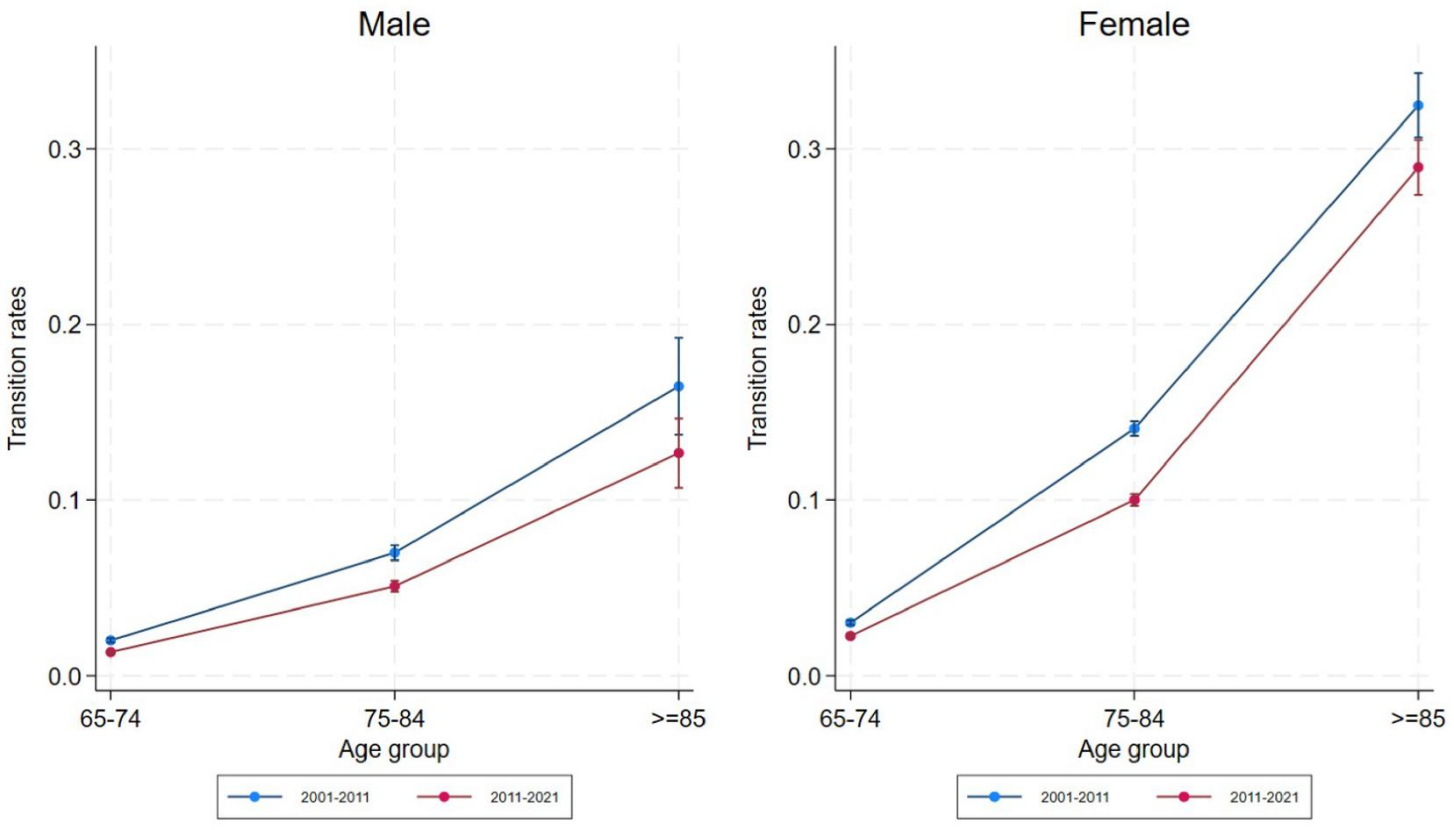
Proportion of older people living in private households moving into a care home after 10 years by sex, age groups, and years, 2001–2021. *Data source*: ONS-LS.

Figure 1 shows transition rates from private households to care homes in 2001−2011 and 2011−2021 by sex and age in England. Overall, the transition rates increase with age, and women consistently show higher rates than men in both periods. Over the two decades, the rate of transitions from private households to care homes declined from 5.5% to 4.0%, with a similar downward trend across all age groups for both sexes. Generally, women experienced a more marked decline than men, with the transition rates decreasing by 1.8 percentage points (vs .9 percentage points for men). The older age groups experienced a sharper decline than the youngest one.

Table 1 presents changes in needs, predisposing, and enabling factors between 2001−2011 and 2011−2021. Regarding predisposing factors, the proportion of men, migrants, and people from ethnic minoritized groups among people aged 65 and above slightly increased from 2001−2011 to 2011−2021. The proportion of people providing intensive care (50 hr and more per week) rose from 4.5% to 5.4%. Enabling factors also shifted: educational attainment significantly improved, with those holding a degree or higher increasing from 15.5% to 19.2%. More people owned their homes outright, and the proportion of people not owning a property fell by 5.5 percentage points. The proportion of married people increased, while the share of those living alone declined, suggesting greater availability of informal care. In contrast, the average number of care home beds per 100 people aged 75 and above dropped from 10.8 to 9.4, indicating a decline in care home supply. Last, self-reported health improved overall, with fewer people reporting bad or very bad health, and those reporting very good health increasing from 7.6% to 12.1%, suggesting reduced levels of population care need.

**Table 1:** Descriptive statistics.

Variables	**2001**–**2011 (*n *= 40,304)**	**2011**–**2021 (*n *= 48,818)**	Total (*n *= 89,122)	*p*
**Care home residence**				
Private households	94.5 (38,099)	96.0 (46,858)	95.3 (84,957)	<.001
Care home	5.5 (2,214)	4.0 (1,940)	4.7 (4,154)	
**Predisposition factors**				
Age				.033
65–74	71.7 (28,901)	71.9 (35,098)	71.8 (63,999)	
75–84	26.2 (10,562)	25.8 (12,593)	26.0 (23,155)	
≥85	2.1 (841)	2.3 (1,127)	2.2 (1,968)	
Sex				<.001
Male	41.2 (16,610)	43.9 (21,427)	42.7 (38,037)	
Female	58.8 (23,694)	56.1 (27,391)	57.3 (51,085)	
Migration status				<.001
UK born	90.5 (36,483)	88.2 (43,034)	89.2 (79,517)	
Migrant	9.5 (3,821)	11.8 (5,784)	10.8 (9,605)	
Ethnicity				<.001
White	90.5 (36,483)	88.2 (43,034)	89.2 (79,517)	
Minorities	9.5 (3,821)	11.8 (5,784)	10.8 (9,605)	
Household type				<.001
Not partnered	29.1 (11,729)	25.5 (12,471)	27.2 (24,200)	
Live with partner only	55.0 (22,167)	57.7 (28,189)	56.5 (50,356)	
Live with both partner and children	7.1 (2,881)	7.9 (3,833)	7.5 (6,714)	
Live with children only	3.2 (1,295)	3.1 (1,512)	3.1 (2,807)	
Other household type	5.5 (2,232)	5.8 (2,813)	5.7 (5,045)	
Marital status				<.001
Not married	37.2 (15,003)	33.7 (16,428)	35.3 (31,431)	
Married/civil partnership	62.8 (25,301)	66.3 (32,390)	64.7 (57,691)	
Unpaid care status				<.001
No	85.7 (34,546)	83.7 (40,859)	84.6 (75,405)	
1–19 hr	8.3 (3,355)	8.9 (4,342)	8.6 (7,697)	
20–49 hr	1.4 (571)	2.0 (971)	1.7 (1,542)	
50+ hr	4.5 (1,832)	5.4 (2,646)	5.0 (4,478)	
**Enabling factors**				
Education				<.001
No qualification	66.5 (26,822)	51.1 (24,931)	58.1 (51,753)	
Lower or other qualification	17.9 (7,234)	29.8 (14,537)	24.4 (21,771)	
University degree	15.5 (6,248)	19.2 (9,350)	17.5 (15,598)	
Tenure				<.001
Own outright	68.8 (27,737)	73.3 (35,773)	71.3 (63,510)	
Partly own	9.9 (3,993)	10.7 (5,220)	10.3 (9,213)	
Not own	21.3 (8,574)	16.0 (7,825)	18.4 (16,399)	
**Need factors**				
Health				<.001
Very good	7.6 (3,073)	12.1 (5,912)	10.1 (8,985)	
Good	31.5 (12,694)	36.9 (17,993)	34.4 (30,687)	
Fair	42.0 (16,933)	35.4 (17,261)	38.4 (34,194)	
Bad	14.5 (5,828)	11.9 (5,822)	13.1 (11,650)	
Very bad	4.4 (1,776)	3.7 (1,830)	4.0 (3,606)	
**Social care resources**				
Care home beds %, mean (*SD*)	10.8 (1.9)	9.4 (1.7)	10.0 (1.9)	<.001

*Note*. *SD* = standard deviation. Percentages with frequencies in parentheses are reported for all variables except care home beds %, for which means and *SD* are reported. To compare the differences in variable distribution across two periods, *t*-tests and $\chi^{2}$ tests were conducted for continuous variables and categorical variables, respectively. The resulting *p*-values are reported in the last column.

*Data source*: ONS-LS and CQC.

### Predictors of moving into a care home in 2001−2011 and 2011−2021

Table 2 presents the results of the logistic regression of the likelihood of moving into a care home in 2001−2011 and 2011−2021. The effects of most predisposing, need, and enabling factors remained largely stable over the two periods. Specifically, the need factor was the strongest predictor of moving into a care home: the likelihood of moving into a care home rose sharply as health declined. Compared with people in very good health, those in very bad health had 16-fold higher odds of moving into a care home in 2001−2011 and 10-fold higher odds in 2011−2021.

**Table 2:** Logistic regression of care home use by year, 2001–2021.

Variables	**2001**–**2011**		**2011**–**2021**	
	Model 1 (*n *= 40,304)		Model 2 (*n *= 48,818)	
	OR	(*SE*)	OR	(*SE*)
**Predisposition factors**				
Sex (ref: Men)	1.429***	(0.075)	1.606***	(0.100)
Age (ref: 65–74)				
75–84	3.555***	(0.199)	3.306***	(0.205)
≥85	9.789***	(0.957)	9.798***	(1.075)
Migration status (ref: UK born)	0.827	(0.100)	1.035	(0.088)
Ethnicity (ref: White)	0.554**	(0.105)	0.333***	(0.060)
Household type (ref: not partnered)				
Live with partner only	0.871	(0.120)	0.803*	(0.083)
Live with both partner and children	0.446***	(0.096)	0.455***	(0.091)
Live with children only	0.514***	(0.067)	0.391***	(0.063)
Other household type	0.887	(0.093)	0.681**	(0.088)
Marital status (ref: not married)	0.694**	(0.091)	0.750**	(0.076)
Unpaid care status (ref: no)		0.940		0.891
1–19 hr	0.940	(0.099)	0.891	(0.086)
20–49 hr	1.165	(0.225)	1.063	(0.200)
50+ hr	1.008	(0.112)	1.411***	(0.129)
**Enabling factors**				
Education (ref: no qualification)				
Lower or other qualification	0.578***	(0.049)	0.730***	(0.047)
University degree	0.836*	(0.069)	0.839*	(0.064)
Tenure (ref: own outright)				
Partly own	0.745**	(0.081)	0.851^+^	(0.079)
Not own	1.248***	(0.068)	1.105^+^	(0.066)
**Need factors**				
Health status (ref: very good)				
Good	3.148***	(0.735)	2.738***	(0.456)
Fair	7.187***	(1.682)	6.678***	(1.133)
Bad	10.894***	(2.649)	9.404***	(1.693)
Very bad	16.427***	(4.155)	9.865***	(2.156)
**Social care resources**				
Care home beds %	1.071***	(0.014)	1.079***	(0.016)
Constant	0.003***	(0.001)	0.003***	(0.001)

*Note*. *n* = observations. Odds ratios (OR) are presented with standard errors (*SE*) in parentheses.

*Data source*: ONS-LS and CQC.

*** *p *< .001.

** *p *< .01.

* *p *< .05.

+ *p *< .1.

Enabling factors also played an important role. People with a university degree and those with lower or other qualifications were less likely to move into a care home than those without. Married people or people living with a partner and/or children were less likely to move into a care home. By contrast, living in a local authority with a greater supply of care home beds was associated with a higher likelihood of moving into a care home. Regarding predisposing factors, being male, younger age, and being from an ethnic minoritized background were associated with a lower likelihood of moving into a care home in both periods.

We also observed changes in the associations between the likelihood of care home entry and some predictors over time. In 2001−2011, individuals who did not own a property had a 24.8% greater likelihood of care home entry compared with those who owned their property outright, while the odds for people who partly owned a property were 25.5% lower. These associations were no longer statistically significant in 2011−2021. Last, intensive informal carers were 41.1% more likely to move into a care home themselves in 2011−2021, an association not observed in the earlier decade.

### Decomposition of the decline in care home admissions

Table 3 presents the results of the decomposition analysis explaining the decline in care home admissions. The aggregate decomposition focuses on the overall contribution of differences in characteristics of the population between the two periods (compositional effects) and differences in the association between these characteristics and moving into a care home (changing coefficient effects) to the declining trend. The results suggest that the decline was mainly due to changes in the composition of need, predisposing, and enabling factors in the population, accounting for only 85.3% of the decline. The contribution of the coefficient effect is relatively small, only taking up 14.7%, suggesting that the relationships between care home admission and these three types of factors remained largely stable over time.

**Table 3:** Non-linear decomposition (Yun’s method) of the decline in care home use from 2001 − 2011 to 2011–2021.

Variables	Composition effects		Coefficient effects	
	Coef.	%	Coef.	%
**2001–2011**	.055***			
**2011–2021**	.040***			
**Difference**	.015***			
**Total**	.013***	85.3	.002	14.7
**Predisposition factors**				
Age	−.000	0.0	−.001	−5.4
Sex	.000***	2.6	−.001	−9.4
Ethnicity	.001***	6.2	−.038	−248.8
Migration status	.000	0.5	.015	95.9
Household type	.000**	2.4	−.009	−56.3
Unpaid care status	−.000	−0.5	.006	4.5
Marital status	.000***	2.7	−.002	−12.1
**Enabling factors**				
Tenure	.000***	2.6	.002	13.1
Education	.002***	12.7	.004	28.1
**Need factors**				
Health	.005***	34.2	−.009	−61.5
**Social care resources**				
Care home bed %	.003***	22.0	−.013	−86.1
Constant			.048	317.0
Observations	89,122			

*Note*. Robust confidence interval in parentheses; *SE* clustered at the local-authority level.

*Data source*: ONS-LS and CQC.

*** *p *< .001.

** *p *< .01.

The detailed decomposition focuses on the contribution of individual factors. Regarding the compositional effects (the first two columns in Table 3), the primary driver for the declining care home admissions was improvement in population health, explaining 34.2% of the decrease. Declining care home beds in local authorities, improvement in educational attainment, and an increasing proportion of people with ethnic minority backgrounds were also key contributors, accounting for 22.0%, 12.7%, and 6.2% of the change, respectively. Additional significant contributors include a reduced proportion of people living alone or unmarried, a decreasing sex ratio among older people, and an increasing proportion of people owning their homes. Regarding the changing coefficient effects, there were no significant contributors after controlling for other factors.

### Robustness checks

We conducted several robustness checks and heterogeneity analyses. First, alternative decomposition approaches, including the traditional Kitagawa–Oaxaca–Blinder linear decomposition (Table S3) and the Fairlie nonlinear decomposition (Fairlie, 2005) (Table S4), yielded consistent findings, with the Fairlie method showing an even larger contribution from health improvements. Second, alternative measures of care needs, including health at the start of each period (see Table S5) and long-term illness (see Table S6), did not change the conclusion that improving health is the primary driver of the declining trends. Third, sex-stratified analyses (see Table S7) showed largely comparable patterns, though some sex differences emerged. For both periods, living with children was associated with a lower likelihood of moving to a care home for women, but not for men.

## Discussion

Using novel data linkages between CQC data and ONS-LS data spanning three decades, this study represents the first longitudinal analysis of *both* demand- and supply-side changes shaping care home use in England. The rates of transition from private households to care homes fell from 5.5% in 2001−2011 to 4.0% in 2011−2021. Decomposition analysis revealed this decline to be largely explained by changes in population characteristics, mainly decreasing needs, changing demographics and household structures, and reduced supply of institutional care resources, rather than changes in how these characteristics predict admission.

A key finding of our study is that improvements in self-reported health are the strongest contributor to the decline in care home use, confirming evidence from previous studies (Wrotek & Kalbarczyk, 2023). This appears to be a positive message for policymakers, demonstrating that an LTC policy framework foregrounding the role of supporting healthy aging and preventive services seems to be effective in delaying people’s entry into care homes and containing the financial costs of LTC service provision. However, this optimism is tempered by several caveats. Health measures in the Census are limited and lack granularity. Both general health and long-term health problems and disabilities are self-reported using broad response categories, which may not accurately capture changes in underlying care needs within the population. More importantly, because our analytical sample only consists of survivors in each period, selective mortality may influence our results. Specifically, the COVID‑19 pandemic, alongside delayed care home entry and tightened eligibility criteria for publicly funded care, may have contributed to a higher mortality rate among those who entered or would have entered care homes during 2011−2021 (Espuny Pujol et al., 2021; Schultze et al., 2022). Given that these people may have died before the end of the observation period, rates of care home entry in the later period may therefore be underestimated. As Table S8 shows, people with poorer health were disproportionately more likely to die before the 10‑year period, especially during 2011−2021. This suggests that survivorship bias could overestimate the contribution of health improvements to the decline in care home entry. Therefore, this finding may not accurately reflect future health trends and should be interpreted with caution.

Our finding that health improvements drive declining institutional care contrasts with two studies conducted in the Netherlands, which found that the decline in institutional LTC was entirely explained by other structural changes (e.g., policy, technological advances, and changes in social norms) rather than changes in need for care (Alders et al., 2017; De Meijer et al., 2015). While differences in health measures (disability scores and chronic diseases) and study periods (the two Dutch studies focused on an earlier period, before 2010) partly explain this, the inconsistency might also stem from whether supply-side factors were included. The two Dutch studies did not include any supply-side factors, so the contribution of changes in the supply of care resources was lumped into the “unexplained” component, and they were unable to pin down which structural changes contributed to the decline.

We advance existing studies by including supply-side factors: key contextual enablers of institutional care use. We find that the declining number of care beds in local authorities is another major contributor to the decline in people moving into care homes. Availability of care homes and awareness about their availability are critical factors in older people’s decisions to move into such facilities (Reed et al., 2003). Recent reductions in care home beds locally may further exacerbate barriers to accessing institutional care for those in need. Although our analysis cannot establish causality (i.e., a reduction in supply could itself reflect a falling demand), the possibility that institutional care resources are increasingly unable to meet demand is nonetheless concerning. Evidence of recent delays in hospital discharge in England, where more people are waiting for permanent beds, suggests that current care home resources may already be insufficient (Schlepper et al., 2023).

Two socioeconomic factors, education and housing tenure, also emerged as major predictors of people moving into care homes. Although these factors are typically considered as “enablers” of care service utilization, as they indicate one’s financial resources to pay for care services, our findings suggest a more complex picture. Specifically, degree holders and homeowners were less likely to enter care homes, consistent with existing evidence that institutional care is more common among socioeconomically disadvantaged groups (Abbing et al., 2023; McCann et al., 2012). One explanation is that highly educated people and homeowners may have greater access to resources supporting their independent living, such as home care and housing modifications. Additionally, the means-tested LTC system in England provides more financial support for institutional care to individuals without housing equity and with limited financial means (McCann et al., 2012). As a result, rising education attainment and expanded homeownership among older adults, driven by policies such as “Right to Buy” and “Right to Acquire” since the 1980s, have likely contributed to declining care home admissions.

Last, the shifting ethnic composition of the older population contributes to the changing patterns of LTC arrangements. Compared with White people, people from ethnic minoritized backgrounds are significantly less likely to move into care homes. While earlier studies on institutional care in the UK rarely addressed ethnicity, largely due to the small proportion of older people from ethnic minority backgrounds (Grundy & Glaser, 1997; Grundy & Jitlal, 2007), demographic shifts make the role of ethnicity increasingly relevant. We find that, as postwar migrant cohorts age, their care preferences and arrangements are shaping broader patterns of LTC use. More importantly, the disparity in the likelihood of entering a care home between ethnic groups has widened over the past two decades. Some qualitative studies on informal care have pointed to factors such as multigenerational co-residence, stigma surrounding institutional care, and experiences of discrimination within health and social care settings as potential contributors to lower formal care use (Lasrado et al., 2021). Future studies need to unpack the changing associations of ethnicity and LTC use.

The study has several limitations. First, despite the large sample size, the Census has limited information on factors contributing to care home entry, such as income and wealth, level of disability, preferences and cultural norms around care, and social networks (Alders et al., 2017). Moreover, we considered only the number of care home beds as a supply-side factor, excluding other important contextual variables such as care home quality, staffing levels, development of care technology, and the availability of home care services due to data limitations, all of which affect the changing landscape of LTC and may be reflected in the “changing coefficients” component of our decomposition. Second, the 10-year interval between the two censuses is relatively long. As a result, we were unable to capture individuals who entered care homes and subsequently exited to return to the community between census waves. The lack of information on the timing of moves into care homes also prevents us from examining the impact of length of stay (LOS) on trends in care home utilization. Studies from the Netherlands have shown that LOS in care homes decreased by 8% from 2012 to 2022, which may have contributed to declining demand for care homes (Alders & Schut, 2019; Alders et al., 2025). Future research in the UK could further explore trends in and the impact of LOS on care homes with more suitable data sources. Third, some census questions have changed slightly over time (e.g., ethnicity and education), while other inconsistencies prevented us from using long-term illness and disability. Last, some information for care home residents may be provided by proxy respondents, which could introduce measurement error in certain subjective measures, such as general health.

Despite these limitations, this study has broad policy relevance. Austerity and the long-standing shift in UK LTC policy toward home and community-based services since the 1990s constitute the key contexts of this study. Although we did not directly assess policy impacts, our analysis of trends in care home residence in England sheds light on how policy priorities may shape individual LTC arrangements, particularly when demographic shifts are taken into account. Although more evidence is needed to substantiate the role of health improvements in explaining the trend in care home entry, the consistently negative association between health and care home entry across the two periods suggests that promoting healthy aging remains central to delaying institutionalization. Further research is needed to assess whether the care needs of older adults are being adequately met by the current supply of institutional care, whether home and community-based services sufficiently address gaps created by declining care home use, and whether reduced reliance on institutional care leads to greater reliance on unpaid carers. The UK is not alone in prioritizing home care. Many European countries have adopted similar policies and witnessed reductions in publicly funded institutional care due to policies aiming for deinstitutionalization (e.g., Denmark, Finland, and Sweden) (Spasova et al., 2018). Our findings offer insights for resource allocation and policy development across these settings. With social care budgets under sustained pressure, it is imperative to reform the current social care funding structure. For example, introducing ring‑fenced funding for preventative social care services, such as early intervention to support individuals to maintain independence, health monitoring, and initiatives that promote lifestyle changes to delay the onset of care needs. Meanwhile, provision of home‑ and community‑based care services should be strengthened. Greater investment is needed to improve the quality and capacity of services such as domiciliary care, community nursing, and home adaptations. This would ensure that alternative forms of care can adequately meet people’s needs in the context of declining local institutional care provision.

Another crucial policy question is whether shifts in LTC priorities affect equitable access to care. The growing gap in care home admissions between ethnic groups underscores the need for ongoing monitoring to better understand how policy changes influence disparities in access to LTC. Understanding how these trends evolve is vital for designing more targeted, inclusive, and equitable health and LTC systems.

## Supplementary Material

gbag104_Supplementary_Data

## Data Availability

The data that support the findings of this study are available from the ONS. Restrictions apply to the availability of these data, which were used under license for this study. More detailed information about the LS and how to make use of it can be found on the ONS website at https://www.ons.gov.uk/aboutus/whatwedo/paidservices/longitudinalstudyls.
