# Supplementary material for: Declining demands or constraining supply? Explaining the trend of care home entry in England 2001–2021
Source: J Gerontol B Psychol Sci Soc Sci. 2026 Jun 8;81(8):gbag104. doi: 10.1093/geronb/gbag104 (PMC13379628; doi:10.1093/geronb/gbag104)
Supplement: gbag104_Supplementary_Data [file gbag104_supplementary_data.docx]

***The Journals of Gerontology, Series B: Psychological Sciences and Social Sciences* Supplementary Material: Zhang, Petrillo, Bennett, & Pryce. Declining demands or constraining supply? Explaining the trend of care home entry in England 2001-2021.**

**Supplementary Methods**

**Yun’s (2004) nonlinear decomposition methods**

Given that the dependent variable is a binary variable and its relationships with explanatory variables are non-linear, we employed a nonlinear decomposition method developed by Yun (2004). According to this approach, the differences in the average likelihood of care home use in 2011 ($i=B$) and in 2021 ($i=A$) can be expressed as follows:

$\underline{T}_{A}-\underline{T}_{B}=\left[ \underline{F\left( X_{A}\beta_{A} \right)}- \underline{F\left( X_{B}\beta_{A} \right)} \right]+ \left[ \underline{F\left( X_{B}\beta_{A} \right)}- \underline{F\left( X_{B}\beta_{B} \right)} \right]$,

where $T$ is the likelihood of care home use, $F$ is the logistic distribution function, $X_{i}$is a vector of independent variables and $\beta_{i}$is a vector of coefficients. The first and second components on the right-hand side of the equation represent the aggregated compositional and changing coefficients effects, respectively. For a robustness check, we also conducted the decomposition using a standard Kitagawa–Oaxaca–Blinder decomposition with linear probability models (see Supplementary Tables 3-4).

**Supplementary Table 1: Sample selection process**

| **Inclusion criteria** | **2011-2021** | | **2001-2011** | |
| --- | --- | --- | --- | --- |
|  | **Excluded *N*** | **Remaining *N*** | **Excluded *N*** | **Remaining *N*** |
| Usual residence at t2 & t1 |  | 466,898 |  | 419,660 |
| Live in England at t2 | 25,167 | 441,731 | 23,851 | 395,809 |
| Age ≥ 65 at t1 | 392,717 | 49,014 | 355,200 | 40,609 |
| Live in private household at t1 | 124 | 48,890 | 206 | 40,403 |
| No missing values | 72 | 48,818 | 99 | 40,304 |
| Final sample size |  | 48,818 |  | 40,304 |

*Data source: ONS LS*

Note: t1 is the start of the period; t2 is the end of the period

**Supplementary Table 2: Missing data pattern**

| **Variable** | **No. of missing values** |
| --- | --- |
| Care home residence (2021) | 72 |
| Care home residence (2011) | 74 |
| Age (2001) | 15 |
| Ethnicity (2001) | 25 |
| Household type (2001) | 25 |
| Marital status (2001) | <10 |
| Tenure (2001) | 25 |
| Unpaid care status (2001) | <10 |

*Data source: ONS LS and CQC*

**Supplementary Table 3: Kitagawa–Oaxaca–Blinder decomposition of the decline in care home use from 2011 and 2021 for robustness check (linear model)**

| **Variables** | **Composition effects** | | **Coefficient effects** | |
| --- | --- | --- | --- | --- |
|  | **Coef.** | **%** | **Coef.** | **%** |
|  |  |  |  |  |
| 2011 | 0.055*** |  |  |  |
| 2021 | 0.040*** |  |  |  |
| difference | 0.015*** |  |  |  |
| Total | 0.012*** | 80.5% | 0.003+ | 19.5% |
| **Predisposition factors** |  |  |  |  |
| Age | -0.000 | -1.6% | -0.011 | -75.3% |
| Sex | 0.000*** | 2.8% | -0.000 | -0.1% |
| Ethnicity | 0.001*** | 4.1% | -0.004 | -28.0% |
| Migration status | 0.000 | 0.6% | 0.004+ | 29.4% |
| Household Type | 0.001*** | 4.3% | -0.000 | -0.1% |
| Unpaid care status | -0.000 | -0.1% | 0.002 | 13.6% |
| Marital status | 0.000** | 2.2% | -0.001 | -4.5% |
| **Enabling factors** |  |  |  |  |
| Tenure | 0.001*** | 3.8% | -0.000 | -0.6% |
| Education | 0.002*** | 11.7% | 0.001 | 6.8% |
| **Need factors** |  |  |  |  |
| Health | 0.004*** | 25.8% | -0.007*** | -48.2% |
| **Social Care resources** |  |  |  |  |
| Care home bed % | 0.004*** | 26.8% | 0.006 | 38.2% |
| Constant |  |  | 0.013 | 88.2% |
|  |  |  |  |  |
| Observations | 89,122 |  |  |  |

Note: SE clustered at the LA level

*** p<0.001, ** p<0.01, * p<0.05, + p<0.1

*Data source: ONS LS and CQC*

**Supplementary Table 4: Non-linear decomposition (Fairlie’s method) of the decline in care home use from 2001-2011 to 2011-2021.**

| **Variables** | **Composition effects** | | **Coefficient effects** | |
| --- | --- | --- | --- | --- |
|  | **Coef.** | **%** | **Coef.** | **%** |
|  |  |  |  |  |
| 2011 | 0.055 |  |  |  |
| 2021 | 0.040 |  |  |  |
| difference | 0.015 |  |  |  |
| Total | 0.013 | 85.9% | 0.002 | 14.1% |
| **Predisposition factors** |  |  |  |  |
| Age | -0.007*** | -46.9% |  |  |
| Sex | 0.001*** | 7.3% |  |  |
| Ethnicity | 0.001*** | 4.4% |  |  |
| Migration status | 0.000 | 0.0% |  |  |
| Household Type | 0.001** | 5.6% |  |  |
| Unpaid care status | -0.000 | -0.3% |  |  |
| Marital status | 0.000 | 3.0% |  |  |
| **Enabling factors** |  |  |  |  |
| Tenure | 0.001*** | 6.1% |  |  |
| Education | 0.003*** | 17.0% |  |  |
| **Need factors** |  |  |  |  |
| Health | 0.009*** | 60.3% |  |  |
| **Social Care resources** |  |  |  |  |
| Care home bed % | 0.004*** | 29.3% |  |  |
| Constant |  |  |  |  |
|  |  |  |  |  |
| Observations | 89,122 |  |  |  |

Note: SE clustered at the LA level

*** p<0.001, ** p<0.01, * p<0.05, + p<0.1

*Data source: ONS LS and CQC*

**Supplementary Table 5: Non-linear decomposition of the decline in care home use from 2001-2011 to 2011-2021 (Robustness check: using health status at the start of the period rather than the end of the period)**

| **Variables** | **Composition effects** | | **Coefficient effects** | |
| --- | --- | --- | --- | --- |
|  | **Coef.** | **%** | **Coef.** | **%** |
|  |  |  |  |  |
| 2011 | 0.055*** |  |  |  |
| 2021 | 0.040*** |  |  |  |
| difference | 0.015*** |  |  |  |
| Total | 0.013*** | 86.8% | 0.002 | 13.2% |
| **Predisposition factors** | |  |  |  |
| Age | 0.000 | 0.1% | 0.001 | 7.3% |
| Sex | 0.000*** | 3.0% | -0.000 | -1.5% |
| Ethnicity | 0.001*** | 7.1% | -0.008 | -54.4% |
| Migration status | 0.000 | 0.5% | 0.003 | 18.5% |
| Household Type | 0.000** | 3.0% | -0.002 | -10.1% |
| Unpaid care status | -0.000 | -0.5% | 0.001 | 9.4% |
| Marital status | 0.000*** | 3.1% | -0.000 | -1.0% |
| **Enabling factors** |  |  |  |  |
| Tenure | 0.001*** | 3.5% | 0.000 | 1.6% |
| Education | 0.003*** | 18.0% | 0.002 | 11.1% |
| **Need factors** |  |  |  |  |
| Health | 0.003*** | 22.7% | 0.001 | 5.0% |
| **Social Care resources** |  |  |  |  |
| Care home bed % | 0.004*** | 26.5% | -0.005 | -30.1% |
| Constant |  |  | 0.009 | 57.2% |
|  |  |  |  |  |
| Observations | 89,122 |  | 89,122 |  |

Note: SE clustered at the LA level

*** p<0.001, ** p<0.01, * p<0.05, + p<0.1

*Data source: ONS LS and CQC*

**Supplementary Table 6: Non-linear decomposition of the decline in care home use from 2001-2011 to 2011-2021 (Robustness check: using long-term illness rather than self-rated health)**

| **Variables** | **Composition effects** | | **Coefficient effects** | |
| --- | --- | --- | --- | --- |
|  | **Coef.** | **%** | **Coef.** | **%** |
| 2011 | 0.055*** |  |  |  |
| 2021 | 0.040*** |  |  |  |
| difference | 0.015*** |  |  |  |
| Total | 0.023*** | 149% | -0.007*** | -49% |
| **Predisposition factors** |  |  |  |  |
| Age | -0.000 | 0% | 0.000 | 2% |
| Sex | 0.000*** | 2% | -0.000 | -1% |
| Ethnicity | 0.001*** | 5% | -0.005+ | -36% |
| Migration status | 0.000 | 0% | 0.002 | 12% |
| Household Type | 0.000* | 2% | -0.002 | -11% |
| Unpaid care status | -0.000 | 0% | 0.000 | 2% |
| Marital status | 0.000*** | 2% | -0.000 | -2% |
| **Enabling factors** |  |  |  |  |
| Tenure | 0.000*** | 2% | -0.000 | 0% |
| Education | 0.002*** | 12% | 0.000 | 1% |
| **Need factors** |  |  |  |  |
| Long-term illness | 0.016*** | 108% | 0.001 | 3% |
| **Social Care resources** |  |  |  |  |
| Care home bed % | 0.003*** | 17% | -0.001 | -10% |
| Constant |  |  | -0.002 | -11% |
|  |  |  |  |  |
| Observations | 89,122 |  | 89,122 |  |

Note: SE clustered at the LA level

*** p<0.001, ** p<0.01, * p<0.05, + p<0.1

*Data source: ONS LS and CQC*

**Supplementary Table 7: Results of logistic regression of care home use by sex and year, 2001-2021**

| **Variables** | **2001-2011** | | **2011-2021** | |
| --- | --- | --- | --- | --- |
|  | **Men** | **Women** | **Men** | **Women** |
| **Predisposition factors** |  |  |  |  |
| Age (ref: 65-74) |  |  |  |  |
| 75-84 | 2.816*** | 3.978*** | 3.030*** | 3.497*** |
|  | (0.267) | (0.284) | (0.305) | (0.245) |
| ≥85 | 7.375*** | 11.097*** | 6.343*** | 11.224*** |
|  | (1.691) | (1.294) | (1.415) | (1.451) |
| Migration status (ref: UK born) | 0.772 | 0.848 | 0.961 | 1.046 |
|  | (0.166) | (0.109) | (0.195) | (0.106) |
| Ethnicity (ref: White) | 0.655 | 0.502** | 0.490* | 0.271*** |
|  | (0.225) | (0.114) | (0.144) | (0.058) |
| Household type (ref: No partnered) |  |  |  |  |
| Live with partner only | 0.673 | 0.986 | 0.735+ | 0.837 |
|  | (0.182) | (0.197) | (0.120) | (0.107) |
| Live with both partner and children | 0.444* | 0.399** | 0.379*** | 0.506** |
|  | (0.158) | (0.124) | (0.108) | (0.129) |
| Live with children only | 0.607 | 0.511*** | 0.430 | 0.402*** |
|  | (0.210) | (0.073) | (0.268) | (0.064) |
| Other household type | 0.808 | 0.899 | 0.521* | 0.752+ |
|  | (0.172) | (0.118) | (0.133) | (0.117) |
| Marital status (ref: not married) | 0.676 | 0.693+ | 0.669* | 0.791+ |
|  | (0.177) | (0.142) | (0.117) | (0.097) |
| Unpaid care status (ref: No) |  |  |  |  |
| 1-19 h | 0.851 | 0.983 | 0.945 | 0.868 |
|  | (0.162) | (0.118) | (0.179) | (0.104) |
| 20-49 h | 1.497 | 1.010 | 1.424 | 0.878 |
|  | (0.428) | (0.269) | (0.445) | (0.190) |
| 50 + | 1.012 | 0.997 | 1.879*** | 1.198 |
|  | (0.196) | (0.150) | (0.291) | (0.153) |
| **Enabling factors** |  |  |  |  |
| Education (ref: no qualification) |  |  |  |  |
| Lower or other qualification | 0.544*** | 0.604*** | 0.551*** | 0.833* |
|  | (0.075) | (0.064) | (0.054) | (0.059) |
| University degree | 0.849 | 0.855+ | 0.727* | 0.905 |
|  | (0.121) | (0.080) | (0.096) | (0.089) |
| Tenure (ref: Own outright) |  |  |  |  |
| Partly own | 0.724+ | 0.759* | 0.836 | 0.852 |
|  | (0.126) | (0.099) | (0.143) | (0.104) |
| Not own | 1.482*** | 1.178** | 1.102 | 1.099 |
|  | (0.144) | (0.072) | (0.133) | (0.074) |
| **Need factors** |  |  |  |  |
| Health status (ref: very good) |  |  |  |  |
| Good | 6.211** | 2.616*** | 3.270** | 2.594*** |
|  | (3.738) | (0.620) | (1.269) | (0.459) |
| Fair | 15.058*** | 5.821*** | 9.370*** | 6.016*** |
|  | (8.886) | (1.354) | (3.383) | (1.146) |
| Bad | 28.856*** | 8.090*** | 15.879*** | 7.808*** |
|  | (17.155) | (1.931) | (6.059) | (1.538) |
| Very bad | 39.004*** | 12.709*** | 13.967*** | 8.848*** |
|  | (23.757) | (2.981) | (5.928) | (2.118) |
| **Social Care resources** |  |  |  |  |
| Care home beds % | 1.076** | 1.068*** | 1.086* | 1.077*** |
|  | (0.030) | (0.018) | (0.038) | (0.022) |
|  | 0.544*** | 0.604*** | 0.551*** | 0.833* |
| Constant | 0.001*** | 0.005*** | 0.002*** | 0.004*** |
|  | (0.001) | (0.001) | (0.001) | (0.001) |
|  |  |  |  |  |
| Observations | 16,608 | 23,693 | 21,427 | 27,391 |

Note: Odds ratios are presented with standard errors in parentheses.

*** p<0.001, ** p<0.01, * p<0.05, + p<0.1

*Data source: ONS LS and CQC*

**Supplementary Table 8: 10-year mortality rate among people aged 65 and above living in private households in England by general health status at the start of the period**

| **10-year mortality rate %** | **General health status in 2001** | | | | | | | | | | | | |
| --- | --- | --- | --- | --- | --- | --- | --- | --- | --- | --- | --- | --- | --- |
|  | **Good** | | | **Fairly good** | | | | | **Not good** | | | | **Total** |
| 2001-2011 | 29.46 | | | 43.54 | | | | | 64.45 | | | | 43.25 |
|  | **General health status in 2011** | | | | | | | | | | | | |
|  | **Very good** | **Good** | | | **Fair** | | **Bad** | | | **Very bad** | | **Total** | |
| 2011-2021 | **19.58** | **29.12** | | | **50.23** | | **66.28** | | | **78.36** | | **41.08** | |
|  |  | |  |  | |  | |  |  | |  | |  |

Note: The 10-year mortality rate was calculated by dividing the number of deaths occurring between two censuses (April 2001- March 2011 and March 2011- March 2021, respectively) by the total number of individuals aged 65 and over living in private households in England at the start of the observation period. It should be noted that the response categories of general health status in the Census 2001 were different from those of the Census 2011 and the Census 2021.

*Data source: ONS LS*
